# Serum Lipidomic Profiling in Breast Cancer to Identify Screening, Diagnostic, and Prognostic Biomarkers

**DOI:** 10.1089/biores.2018.0022

**Published:** 2020-01-03

**Authors:** Karima Fichtali, Amine Bititi, Adil Elghanmi, Bouchra Ghazi

**Affiliations:** ^1^ Cheikh Khalifa International Hospital, Faculty of Medicine, Mohammed VI University of Health Sciences (UM6SS), Casablanca, Morocco.; ^2^ National Laboratory of Reference, Faculty of Medicine, Mohammed VI University of Health Sciences (UM6SS), Casablanca, Morocco.

**Keywords:** biomarkers, breast cancer, lipidomics, prognosis, serum lipid profile, statin

## Abstract

Breast cancer is the major mortality cause of women worldwide. In the course of management of breast cancer, the identification of a biomarker is important in enhancing our knowledge on cancer pathology, predicting the response to treatment, and selecting the patients who are more favorable to receive certain treatments. These biomarkers have a prognostic value. In addition to traditional breast cancer prognosis factors such as the tumor size and grade, the axillary lymph node micrometastasis, and biomarkers such as HER2/neu, newly discovered biomarkers have been discovered. Some of these factors are genetic signature in tissue or in peripheral blood. Lipid profil, a simple and accessible biological examination, has been a novel path on the prediction of breast cancer risk of occurrence and recurrence in many studies. The main goal of our review is to evaluate lipid profile and breast cancer risk with an emphasis on the prognosis value of lipid profiles in breast cancer patient management.

## Introduction

Nowadays, breast cancer is the most commonly diagnosed life-threatening cancer in women and remains the leading cause of death by cancer among women, with 1.7 million cases and 521,900 deaths estimated in 2012.^1^ Indeed, breast cancer accounts for 25% of all cancer cases and 15% of all cancer deaths in women.^1^ In a developing country, about 55.6% of all cancer cases and 62.1% of deaths are due to breast cancer.^2^ Interestingly, breast cancer incidence rates vary substantially by world region.^3^ Although there is a lower incidence of breast cancer in the North Africa region compared to Western countries, the global picture in Northern Africa is dynamically changing.^4^ Morocco, for example, is currently a country in epidemiological transition, and the breast cancer is becoming one of the main public health problems.^5^

The rising incidence tendency in low- and middle-income countries is mainly due to increases of risk factors caused by economic development and urbanization. Many factors among others include overweight and obesity, physical inactivity, earlier age at menarche, delayed childbearing, and/or having fewer children, as well as shorter duration of breastfeeding.^6–8^ Early screening, diagnosis, and management, together with improved treatments, may explain the international disparities in breast cancer mortality trends. Overall, there was an upward trend in the prevalence of overweight among Moroccan women, which increased over a period of 1 year from 29.0% in 1999 to 47.8% in 2000.^9^ In Morocco, remarkable change in breast cancer incidence is largely due to improved diagnostic facilities and health care accessibility, as well as raising awareness of breast cancer.

The advanced stage at diagnosis in developing countries is contributed mainly to the lack of access to mammography, availability and quality of primary health care facilities, and the psychological and social impacts of treatments particularly for mastectomy.^10,11^ It is noteworthy that in 2003, a study conducted by the World Health Organization revealed that only 2.1% of Moroccan women aged 40 years or more had a clinical breast examination (or mammogram) in their life (Ministry of health, Morocco, 2007).

Nowadays, it is well established that the prognosis of most cancers is determined by the cancer stage at diagnosis, and women with early stage breast cancer have higher survival rates.^10,12^ Unfortunately, in Morocco like most developing countries, a very high proportion of breast cancer cases are diagnosed at late stages.^13–16^ The trends are completely different in western countries, in which the majority of patients with breast cancer are diagnosed at an early stage. For example, in the United States, it reached a high of 80% of cases.^17^

## Lipid Biomarkers in Breast Cancer

The development and progression of cancer is a very complex multistep process involving reciprocal interactions of behavioral and environmental risk factors, dysregulated lipid homeostasis, and the cancer microenvironment.^18–20^

Blood lipids, used for both adipose and glucose transportation, and lipoproteins, used for membrane production, have been incriminated in carcinogenesis through insulin resistance, inflammation, oxidative stress pathways, and the generation of signaling molecules in cancer cells.^20–23^ It has been shown that healthy lifestyle modifications for cancer prevention such as dietary changes,^24,25^ exercise,^26^ and smoking cessation^27^ are effective to increase high-density lipoprotein (HDL) cholesterol (i.e., “good” cholesterol).

Dyslipidemia, a major component of metabolic syndrome, has been reported as playing a considerable role in the carcinogenesis of different cancers, like prostate cancer, ovarian cancer, and kidney cancer.^28–30^ Few well-documented metabolomics and lipidomics studies in breast cancer have shown the relation of lipids and breast cancer diagnosis and progression.^31–33^ Nevertheless, the focus of most of these studies has been total levels of lipids in cancer patients, and only a few of them were conducted with patients with benign breast diseases. In a recent comprehensive evaluation of plasma lipid profiles performed by Yang et al., including benign breast disease patients, it has also indicated the diagnostic efficiency of the lipid markers in breast cancer diseases.^34^

Presently it is well known that lipids are implicated in various ways in the pathogenesis of several human diseases, including breast cancer.^35^ In particular, the oncological potential of complex polar lipids may participate in development and metastasis of breast cancer.^36^ Recently, a panel of 15 plasma lipid species was identified as potential biomarkers for early diagnosis of breast cancer and was able to distinguish the early stage of breast cancer from benign lesions.^37^

Triglyceride levels, reflecting insulin and glycemia status, may also express the complex indirect effects of hyperglycemia and/or hyperinsulinemia on tumor-promoting pathways.^38^ The mechanism for the insulin-cancer link has been hypothesized to be mainly related to increased production of free insulin-like growth factor-1, a potent mitogen, and adipocyte-derived vascular endothelial growth factor, a key factor in tumor angiogenesis that leads to cell survival and migration.^38,39^

Relatively sparse studies have evaluated the relationship between novel lipid biomarkers, such as apolipoprotein A-I (Apo A-I) and apolipoprotein B-100 (Apo B-100), and cancer risk.^18^ Apo A-I, the major protein components of HDL cholesterol, and Apo B-100, the major protein components of low-density lipoprotein (LDL) cholesterol, manifest the antiatherogenic and proatherogenic effect of HDL and LDL cholesterol, respectively.^18^ The prospective Swedish Apolipoprotein Mortality Risk study has shown no significant association of Apo B with colorectal cancer, but a significant positive association between triglycerides and colon cancer risk was found.^40^ There is no consistent information with regard to Apo A-I and breast cancer because studies are scarce with mixed results.^41,42^ In this issue, Han et al. showed that high Apo A-I levels were associated with higher risk of breast cancer.^41^ In the opposite, Chang et al. revealed a reduced risk of breast cancer with higher Apo A-I.^42^ In a large nested case–control study, measurement of plasma lipid marker concentrations at multiple times before the diagnosis of breast cancer showed decreased risk in breast cancer with higher Apo A-I.^43^

## Lipids as Early Diagnostic Biomarkers

Early diagnosis plays an important role in the prognosis of patients with breast cancer. Mammography is the most reliable tool used for breast cancer scanning with the sensitivity at 54% to 77%.^44^ However, the outcome of this screening tool is often not satisfactory because of the false-negative results and high false positive rate.^45^ In screening mammography, the over-diagnosis rate of breast cancer is variably quoted between 10% and 40%.^46^ Hence, abnormal screening mammograms engender additional expensive diagnostic tests, magnetic resonance imaging (MRI) and tissue sampling (fine needle aspiration, core biopsy, or excisional biopsy). Moreover, MRI has a high false-positive rate in differentiating breast lesions and may lead to a high frequency of useless biopsies of benign lesions, causing needless anxiety, physiological distress, and additional costs for patients.^47^ What worsens the situation is that about 10% of patients will be called back, from each screening examination for additional testing, but only 5% will be diagnosed with breast cancer.^48^ Thus, there is a pressing need for high accuracy technique and early diagnosis of malignant breast lesions, particularly for differentiating early cancer from benign lesions to avoid irrelevant, expensive, and invasive screening for benign patients.

Blood-based tumor markers are one approach to address these challenges. However, serum tumor markers are not yet used in clinical trials; for example, CA15.3 and BR27.29 are not used for breast cancer detection for their low sensitivity.^49–52^ In this issue, Min et al., performed the analysis on four different categories of phospholipids (phosphatidylserine [PS], phosphatidylinositol [PI], phosphatidylglycerol, and phosphatidic acid), from urine of breast cancer patients.^31^ Compared to healthy controls, in breast cancer patient group, two PS molecules (18:1/18:1 and 18:2/18:0) showed a significant increase; postoperative (after surgery) concentrations were reduced to normal levels. However, PI molecules (18:0/20:4) were significantly decreased in breast cancer samples.^31^ Chen et al., have identified a panel of plasma lipid species able to differentiate the early stage of breast cancer from benign lesions and act as relevant biomarkers for early diagnosis of breast cancer.^37^ This lipid profile was able to generate fast, high-efficiency, and high-throughput detection and involved only a minimally invasive procedure.

Lipidomics, a novel detection method, may serve as a promising prognostic evaluation of breast cancer especially in high-risk patient groups. Chen et al., have identified 15 lipid subtypes revealing significant differences of plasma concentration between breast cancer and benign patients.^37^ The plasma concentrations of phosphatidylcholine (PC) and ether-linked PC classes were observed to increase in patients with breast cancer.^37^ Exosomes derived from cells are rich in different components, such as heat shock proteins, annexin, lipids, and nucleic acids.^53–55^ Phuyal et al. have reported that an increase in cellular ether lipids (including PCs) affects the release and composition of exosomes in human prostate cancer PC-3 cells.^56^ In cancer patients, decreased levels of lysophosphatidylcholine (LPC) are linked with a high metabolic activity and activated inflammatory processes.^57^ LPCs have also been suggested to be a general indicator of disease severity.^37,57^ In breast cancer patients, the plasma concentration of LPC and cholesterol ester (CE) was noted to decrease, compared to that found in benign patients.^37^ The lower levels of LPCs seem to be associated with a higher metabolism rate in breast cancer patients. The repercussion of CE in breast cancer still has to be elucidated despite the association reported between CE and poor clinical outcome in human breast cancer.^58^

## Lipid Biomarkers in Prognosis of Breast Cancer

Recent studies have reported the modification of lipid levels in prognosis of different types of cancers.^59,60^ For instance, reduction of cholesterol in oral cancers is alarming for the progression of cancerous lesions.^61^ Opposite results regarding the association between lipid levels and cancer progression seem to be due to the type of tumor and also by pathophysiological role of lipids related to cancer progression.

The association of lipids and lipoproteins with breast cancer was determined.^62^ Assessment of serum lipids among breast cancer, by Ghahremanfard et al., revealed highest levels of cholesterol and lipoproteins.^63^ Thereby, it has been concluded that increased serum lipid levels could have an important role in the progression of cancers in women, including breast and ovarian cancers, while lower level of lipids and lipoproteins may be used as markers for discriminating gastric cancer.

A higher consumption of fats increases circulating estrogen levels, increasing proliferation and cell damage, with direct effects on cancer growth.^64^ It has been found that there is an association between cholesterol and high HDL levels and increased risk of breast cancer.^62,63^ In another study, low HDL was correlated with increased postmenopausal breast cancer risk.^65^ Thereby, the association between serum HDL level and risk of breast cancer seems to be potentially influenced by menopausal status. By that, it has been reported that premenopausal patients have mean HDL levels lower than matched controls, while postmenopausal patients had higher levels than the controls.^66,67^ Interestingly, changes in serum lipid and lipoprotein profile are not only linked with the presence of some types of cancers but also there was a direct correlation between high LDL level and increased risk for lymph node metastasis.^63^

Potential prognostic value of lipidomic profile has been subjected to several investigations. It is commonly known that lipogenesis increased most solid tumors early on, with a potential impact on chemosensitivity of cancer cells.^68^ Hilvo et al. have investigated the association between the lipidomic profiles and pathologic complete response (pCR) in breast cancer patients receiving neoadjuvant chemotherapy.^69^ The results of this study have shown that serum triacylglycerol, containing C18:1 fatty acyl chains, was found at lower concentrations in the patients showing pCR. Formerly, Wei et al. achieved a serum metabolite analysis to identify a potential biomarker in response to neoadjuvant chemotherapy regimens against breast cancer (predictive power).^70^ Thus, metabolomic profiles of patients with pCR revealed significant differences in threonine, glutamine, and isoleucine levels; even more interestingly, in pCR patients, the free linoleic acid (C18:2) was detected in lower levels.^70^

## Statins and Breast Cancer Prognosis

Nowadays, a lot of studies sustain the anticancer effects of HMG-CoA reductase inhibitors (statins). These studies do not support an association between statin use and reduced breast cancer incidence, but does support a protective effect of statins on breast cancer prognosis. Particularly, the simvastatin, a type of statins, may reduce the risk of breast cancer recurrence.^71^ Simvastatin inhibits DNA binding of the NF-kappa-B transcription factor, diminishes the expression of the antiapoptotic protein bcl-xL, and increases transcription of phosphatase and tensin homolog, which represses the oncogenic phosphatidylinositol-3-kinase pathway.^72^ Simvastatin accelerated the production of a variant of the p53 transcription factor and diminished the formation of bone metastases.^73^ This preventive effect would have a cost-effective low-toxicity option for adjuvant therapy for breast cancer survivors.^71^

## Conclusion

Breast cancer prognosis factors are an important component of the management process. They improve the selection of the appropriate treatment for a selected patient. Lipid profile is a novel prognosis factor as shown by multiple studies. It's a promising path in the prevention and better treatment of patients. Actually, medical literature suggests that diet and physical activity could influence survival. Moreover, statins are now used to enhance the positive prognosis of breast cancer. More studies are necessary for further evidence based guidelines.

## Abbreviations Used

Apo A-I: apolipoprotein A-I
Apo B-100: apolipoprotein B-100
CE: cholesterol ester
HDL: high-density lipoprotein
LDL: low-density lipoprotein
LPC: lysophosphatidylcholine
MRI: magnetic resonance imaging
PC: phosphatidylcholine
pCR: pathologic complete response
PI: phosphatidylinositol
PS: phosphatidylserine
